# Aptamer-based Targeted Delivery of a G-quadruplex Ligand in Cervical Cancer Cells

**DOI:** 10.1038/s41598-019-44388-9

**Published:** 2019-05-28

**Authors:** Josué Carvalho, Artur Paiva, Maria Paula Cabral Campello, António Paulo, Jean-Louis Mergny, Gilmar F. Salgado, João A. Queiroz, Carla Cruz

**Affiliations:** 1 CICS-UBI - Centro de Investigação em Ciências da Saúde, Universidade da Beira Interior, Av. Infante D. Henrique, 6200-506 Covilhã, Portugal; 2 Unidade de Gestão Operacional em Citometria, Centro Hospitalar e Universitário de Coimbra (CHUC), Coimbra, Portugal; 3 CIMAGO/iCBR/CIBB, Faculdade de Medicina da Universidade de Coimbra, Coimbra, Portugal; 4 Instituto Politécnico de Coimbra, ESTESC-Coimbra Health School, Ciências Biomédicas Laboratoriais, Coimbra, Portugal; 5 Centro de Ciências e Tecnologias Nucleares, Instituto Superior Técnico, Universidade de Lisboa, Estrada Nacional 10 (km 139,7), 2695-066 Bobadela, LRS Portugal; 6 Univ. Bordeaux, ARNA laboratory, INSERM, U1212, CNRS UMR 5320, IECB, F-33600 Pessac, France; 7 Institute of Biophysics, AS CR, v.v.i. Kralovopolska 135, 612 65 Brno, Czech Republic

**Keywords:** Drug delivery, Oligo delivery

## Abstract

AS1411 is a G-rich DNA oligonucleotide that functions as an aptamer of the protein nucleolin, found at high levels on the surface of cancer cells but not on the surface of normal cells. Herein, we have studied AS1411 as a supramolecular carrier for the delivery of an acridine-based G-quadruplex ligand, C_8_, to HeLa cancer cells. Two AS1411 derivatives, LNA-AS1411 and U-AS1411, were also tested, in an attempt to compare AS1411 pharmacological properties. The results showed that AS1411-C_8_ complexation was made with great binding strength and that it lowered the ligand’s cytotoxicity towards non-malignant cells. This effect was suggested to be due to a decreased internalization of the complexed versus free C_8_ as shown by flow cytometry. The AS1411 derivatives, despite forming a stable complex with C_8_, lacked the necessary tumour-selective behaviour. The binding of C_8_ to AS1411 G-quadruplex structure did not negatively affect the recognition of nucleolin by the aptamer. The AS1411-C_8_ repressed c-MYC expression at the transcriptional level, possibly due to C_8_ ability to stabilize the c-MYC promoter G-quadruplexes. Overall, this study demonstrates the usefulness of AS1411 as a supramolecular carrier of the G-quadruplex binder C_8_ and the potential of using its tumour-selective properties for the delivery of ligands for cancer therapy.

## Introduction

AS1411 is a synthetic 26-base DNA aptamer previously discovered by Bates and co-workers as an agent targeting the nucleolin protein with high affinity and specificity, eliciting a potent antiproliferative effect on a variety of cell lines^1,2^. Nucleolin is a multifunctional protein playing essential roles in cell survival, growth and proliferation. The protein is localized primarily in the nucleus of normal cells, while on cancer cells it is also present in the cytoplasm and at the cell surface^3^. This property confers a tumour-selective behaviour to AS1411 which targets preferentially the external domain of surface nucleolin of cancer cells. This oligonucleotide reached Phase 2b clinical trials for acute myeloid leukaemia and renal cell carcinoma; however, despite a good tolerance and safety profile, the trial was terminated due to suboptimal pharmacokinetics (rapid clearance) and low potency^2^. The mechanism of the cytotoxicity of AS1411 is still under debate and multiple nucleolin-dependent and independent biological effects have been described^2^. Recently, methuosis has been proposed as the likely cell death mechanism induced by AS1411, which is characterized by altered cell morphology and hyperstimulation of macropinocytosis combined with perturbed vesicle trafficking^4^. AS1411 is able to fold into a highly polymorphic G-quadruplex structure which makes it more stable against the serum nucleases and pH fluctuations, with increased cellular uptake efficacy^1,2^.

Regardless of the clinical application of AS1411 being hindered, its tumour-selective properties have increasingly motivated its study as a drug delivery system of therapeutic agents and imaging probes. The variety of applications range from nanoparticle-linked AS1411 formulations to covalent/noncovalent drug conjugates^2^. The diversity of cargoes that have been efficiently delivered by AS1411 extend to G-quadruplex ligands as its own structure is a stable G-quadruplex. Recently, AS1411 was noncovalently bound to porphyrin TMPyP4, in an attempt to increase its delivery to MCF7 breast cancer cells within a photodynamic therapy approach^5^. This strategy was shown to increase the accumulation of TMPyP4 in MCF7 cancer cells when compared to non-malignant cells. Several examples in the literature provide evidence that attaching AS1411 to a wide variety of nanoparticles or other entities is an excellent strategy for delivering these cargoes inside of cancer cells. Moreover, improving AS1411 stability and biological properties, mainly through chemical modification on deoxynucleotides at appropriate positions in AS1411, could also be a strategy to improve the therapeutic function of the aptamer^2^.

Herein, we have evaluated AS1411 and its modified derivatives designated by LNA-AS1411 and U-AS1411 as drug delivery systems to carry an acridine orange-based ligand (C_8_)^6^ to HeLa cervical cancer cells. The ligand C_8_ was previously published and shown to bind and stabilize promoter and telomeric G-quadruplexes with high affinity (*K*_D_ ≈ 10^−7^ M). C_8_ showed cytotoxic activity against HeLa cells with an IC_50_ value around 1 µM at 24 h incubation; however, similar values were observed for non-malignant cells. Therefore, targeted carriers that promote the selective accumulation of C_8_ in cancer cells, while preventing toxicity in healthy surrounding cells, are of crucial importance to improve its pharmacological profile as a potential drug for anticancer therapies. To address this goal, we have studied the formation of supramolecular complexes between C_8_ and AS1411, as well as with its derivatives LNA-AS1411 and U-AS1411. Herein, we also describe the physical properties and biological activities of the resulting C_8_-oligonucleotide supramolecular complexes.

## Materials and Methods

### Oligonucleotides and compounds

AS1411 (5′-GGTGGTGGTGGTTGTGGTGGTGGTGG-3′), LNA-AS1411 (5′-gGTGGTGGTGgTTGTGGTGGTGGTGg-3′, where g stands for LNA-modified guanine), U-AS1411 (5′-GGTGGTGGTGGUUGTGGTGGTGGTGG-3′, where U stands for uracil base), 5′-FAM-AS1411-TAMRA-3′ and Cy5-AS1411 were purchased from Eurogentec (Belgium) with HPLC-grade purification and used without further treatment. Stock solutions of about 1 mM were prepared using Milli-Q water and stored at −20 °C until used. For all experiments, the oligo was annealed by heating to 95 °C for 10 min and cooling down on ice until used. Hoechst 33342 was purchased from Thermo Scientific (USA). Compound 10-(8-(4-iodobenzamide)octyl))-3, 6-bis(dimethylamine) acridinium iodide (C_8_) was synthesized as previously described^7^. The compound was dissolved in DMSO to obtain a 10 mM stock solution and further diluted in Milli-Q water.

### Thermal difference spectra (TDS)

Thermal difference spectra (TDS) were collected on a Thermo Scientific^TM^ Evolution^TM^ 201 UV-Visible Spectrophotometer (Thermo Fisher Scientific, USA). Spectra were obtained in the 220-335 nm range (scan rate of 200 nm/min and 1 nm data intervals) above and below the melting temperature, at 90 °C and 20 °C, respectively^8^. DNA was used at 3 μM concentration in 30 mM phosphate buffer (15 mM KH_2_PO_4_, 15 mM K_2_HPO_4_, pH 7.1) containing 100 mM KCl. The TDS spectrum was calculated by subtracting the spectrum at 20 °C from the spectrum at 90 °C. The following UV TDS factors were used for analysis: ΔA_240_/ΔA_295_, ΔA_255_/ΔA_295_, ΔA_275_/ΔA_295_. The data was normalized relative to the maximum absorbance.

### Circular dichroism

Circular dichroism (CD) experiments was performed on a Jasco J-815 CD spectrometer equipped with a Peltier-type temperature controller (model CDF-426S/15). Spectra acquisition was performed in 1- or 10-mm quartz cuvettes at a DNA concentration of 10 µM in 30 mM potassium phosphate buffer containing 100 mM KCl. Spectral width was set to 220–340 nm, with a scan speed of 100 nm/min, 1 nm bandwidth, 1 s integration time over 4 averaged accumulations. During titrations, the required volume of ligand solution was added to the quartz cell.

For CD melting studies, buffer conditions were adjusted so that the oligos would melt around 50 °C, particularly by using 10 mM lithium cacodylate buffer, pH 7.2, containing 10 mM KCl and 90 mM LiCl. Melting curves were obtained by monitoring a single wavelength between 20 and 110 °C with a heating rate of 2 °C/min. Data were converted into fraction folded (θ) plots, fitted to a Boltzmann distribution using OriginPro 2016 and the melting temperatures (*T*_m_) determined from the two-state transition model.

### Fluorescence studies

Fluorescence titrations were conducted on a Horiba FluoroMax4 fluorometer (Japan) using a high-precision quartz suprasil cuvette (light path 10 × 4 mm). The fluorescence spectra were obtained between 530–700 nm and averaged over three scans. The excitation wavelength was 498 nm, matching the maximum absorbance of C_8_. To study ligand–aptamer complex formation, previously annealed aptamer sequence was titrated into the cuvette containing a solution of 5 μM C_8_ in 30 mM potassium phosphate buffer (pH 7.2) containing 100 mM KCl. Spectra were acquired after a 5 min equilibration period. Fluorescence data was converted into fraction of bound ligand (α) plots, fitted to the saturation binding Hill model (OriginPro 8) and the dissociation constant (*K*_D_) and Hill coefficient (*n*) values determined.

Fluorescence titrations were also carried with nucleolin protein. In this case, labelled Cy3-AS1411 at 1 μM, was titrated with nucleolin in the absence and presence of C_8_ to evaluate the effect of ligand binding in the affinity of the aptamer towards the protein. The fluorescence emission spectra were obtained between 555 and 700 nm and averaged over three scans. The excitation wavelength was 540 nm, matching the maximum absorbance of Cy3.

### Cell viability assay

Normal human dermal fibroblasts (NHDF) were grown in RPMI-1640 medium supplemented with 0.01 M HEPES, 0.02 M L-glutamine, 0.001 M sodium pyruvate, 10% fetal bovine serum, and 1% penicillium/streptomycin antibiotic. Human cervical cancer cells (HeLa) were grown in DMEM medium supplemented with 10% fetal bovine serum, and 1% penicillium/streptomycin antibiotic. Cultures were maintained at 37 °C in a humidified atmosphere containing 5% CO_2_. For MTT assays, cells were seeded in 48-well plates (5 × 10^3^ cells/well) and incubated overnight for cell adhesion. Then, cells were incubated for 7 days with the aptamers at 15 μM, C_8_ at 1 μM and the preformed ligand-aptamer complex using the same compound/DNA concentrations. Complex formation was performed by incubating the aptamers with C_8_ for 10 min prior to incubation. Wells containing untreated cells were used as control. At the end of incubation, the media was replaced with fresh media containing 3-(4, 5-dimethylthiazol-2-yl)-2, 5-diphenyltetrazolium bromide salt (MTT) and further incubated at 37 °C for 1 or 4 h for HeLa and NHDF, respectively. Finally, the formazan crystals were dissolved in DMSO and absorbance was recorded in a Bio-Rad xMark™ microplate reader at 570 nm. Cell viability relatively to control was expressed as mean ± SEM from at least three different experiments. The IC50 values of C_8_ at 7 days incubation period were determined by incubating the cells with different compound concentrations ranging from 0.01 to 2 μM. All data treatment was performed using GraphPad Prism 6.

### Cell membrane damage

The effect of the ligand-aptamer complex on cell membrane integrity was assessed by the lactate dehydrogenase (LDH) assay using Pierce™ LDH Cytotoxicity Assay Kit (Thermo Fisher Scientific, USA). Cells were seeded in 48-well plates (5 × 10^3^ cells/well) and incubated overnight for cell adhesion. Then, cells were incubated for 7 days with the preformed ligand-aptamer complex using the same compound/DNA concentrations used for MTT assay (15 μM aptamer and 1 μM C_8_). After exposure, 50 μL/well of supernatant were transferred into optically clear 96-well flat-bottom microplates and mixed with 50 μL/well of reaction mixture and incubated for 30 min at room temperature. The absorbance of the samples was then measured at 490 nm using a Bio-Rad xMark™ microplate reader. The percentage of cytotoxicity is calculated with the following equation^9^:

$$ \% \,cytotoxicity=\frac{{\rm{Experimental}}\,{\rm{LDH}}\,{\rm{release}}\,({{\rm{OD}}}_{490})}{{\rm{Maximum}}\,{\rm{LDH}}\,{\rm{release}}\,({{\rm{OD}}}_{490})}\times 100$$

Cells treated with lysis buffer were used as positive control (maximum LDH release). The experiments were performed in at least two different plates for each cell line, using triplicate wells for each condition.

### Flow cytometry analysis

Cells were seeded in 12-well plates (5 × 10^5^ cells/well) and incubated overnight for cell adhesion. Then, cells were incubated for 24 h with C_8_ at 1 μM as control and the preformed ligand-aptamer complex using the same compound/DNA concentrations as used in the MTT assays. After the incubation period, the wells were washed by rinsing with PBS three times. Cells were then trypsinized, resuspended in PBS and analysed in a BD FACSCanto™ II flow cytometry system (BD Life Sciences, US) to evaluate the uptake of the aptamer-ligand complex. Non-specific colouring and debris were excluded by analysing FSC *vs* SSC density plot as dead cells and debris have lower forward scatter levels.

### RNA isolation and RT-qPCR for gene expression analysis

To analyse the effect of the ligand-aptamer complex at genetic level, RT-qPCR analysis of c-MYC and nucleolin expression was performed. Cells were seeded in 12-well plates (1 × 10^5^ cells/well) and incubated overnight. Following cell adhesion, these were incubated for 7 days with free C_8_ at 1 μM and free aptamer at 15 μM as control, and the preformed ligand-aptamer complex using the same compound/DNA concentrations. Total RNA was extracted from the cells using TRIzol reagent (Invitrogen, USA). 500 ng of total RNA were reverse transcribed using RevertAid First Strand cDNA Synthesis Kit (Thermo Fisher Scientific, USA), according to the kit instructions. For quantitative analysis of gene expression, RT-qPCR amplification of cDNA was performed using SYBR® Green PCR Master Mix (Thermo Fisher Scientific, USA) on a CFX Connect™ Real-Time PCR Detection System (Bio-Rad, USA). The relative quantification of gene expression was based on the comparative threshold cycle (C_T_) method in which the quantity of transcripts is determined as 2^−(ΔCT target−ΔCT control)^, normalized to levels of β-actin and relative to the untreated control cells. The following forward (Fw) and reverse (Rv) primers were used: c-MYC-Fw 5′-TGAGGAGACACCGCCCAC-3′ and c-MYC-Rv 5′-CAACATCGATTTCTTCCTCATCTTC-3′; NCL-Fw 5′-AGGAGGAGGAAGAAGAGGAG-3′ and NCL-Rv 5′-ACAAAGAGATTGAAAGCCGTAG-3′; β-actin-Fw 5′-AAGAGAGGCATCCTCACCCT-3′ and β-actin-Rv 5′-TACATGGCTGGGGTGTTGAA-3′. The RT-qPCR parameters were the following: an initial denaturation at 95 °C for 3 min, followed by 40 cycles with denaturation at 95 °C for 30 s, annealing at 57 °C for 30 s, and elongation at 72 °C for 30 s, followed by a dissociation stage at 65 °C 5 s. Each sample was run in triplicate from three different experiments and results are expressed in relative levels to respective controls (means ± SEM).

### Fluorescence resonance energy transfer (FRET) melting

Fluorescence resonance energy transfer (FRET) melting experiments were performed on a CFX Connect™ Real-Time PCR Detection System (Bio-Rad, USA). AS1411 was purchased with FAM and TAMRA at the 5′ and 3′ ends, respectively. The fluorescence measurements were performed in 10 mM lithium cacodylate buffer containing 10 mM KCl and 90 mM LiCl as previously reported^10^. Labelled-AS1411 was used at 0.2 µM and C_8_ at 1 µM. For competition experiments with nucleolin, the protein was previously incubated with AS1411 at 1 μM and 2 µM for 30 min at 37 °C. FAM fluorescence intensity was recorded between 25–95 °C, with a temperature increment of 1 °C/min. The excitation and detection wavelengths were 492 and 516 nm, respectively. Each experimental condition was tested in triplicate in at least two separate plates. The melting temperatures were determined from the normalized curves as the temperature for which the normalized emission was 0.5.

### Confocal fluorescence microscopy imaging

HeLa and NHDF cells were cultured in Ibidi 8-well μ-slides (1 × 10^4^ cells/well) overnight at 37 °C in a humidified atmosphere containing 5% CO_2_. After cell adhesion, cells were incubated with 1 μM Cy5-AS1411 in the presence and absence of 0.5 μM C_8_, for 7 days. At the end of each of the 7 days of incubation, the excess of fluorophores was washed off by rinsing with PBS three times. Cell nucleoli were then stained with 1 µM nuclear probe Hoechst 33342 for 15 min. Prior to visualization, excess probe was washed off by rinsing with PBS three times. Images were acquired using a Zeiss AxioObserver LSM 710 microscope with 405, 488 and 564 nm laser excitation for Hoechst 33342, C_8_ and Cy5-AS1411, respectively. Appropriate emission bands were selected for the three fluorescent channels, considering the spectral overlap and potential bleed-through between channels. Images were processed with Zeiss ZEN software. The quantitative analysis of Cy5-AS1411 internalization in the presence and absence of C_8_ was determined as described in literature^11^. In brief, the corrected total cell fluorescence (CTCF) of at least 100 AS1411-positive cells for each experimental condition in three different samples was measured. CTCF can be determined as CTCF = ID − (A × MFB) where the ID, A, and MFB correspond to integrated density, selected cell area, and mean fluorescence of background readings, respectively.

### Statistical analysis

The statistical analysis was performed by using Student’s unpaired t test. A *p* value < 0.05 was considered statistically significant. Data analysis was performed in GraphPad Prism 6 (San Diego, CA, USA).

## Results and Discussion

**Figure 1:**
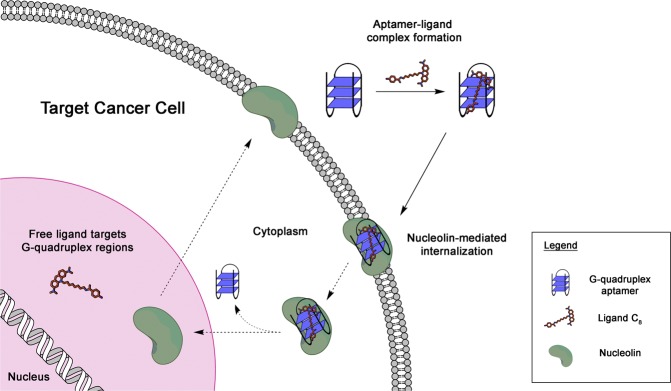
Schematic representation of proposed hypothesis of using AS1411 and its derivatives to carry ligand C_8_ into cancer cells overexpressing nucleolin.

AS1411 aptamer is extensively documented as a potential targeting agent for the development of cancer-selective drug delivery systems. It has been shown before that it can deliver a variety of attached cargoes (both covalently and non-covalently) to various cancer cell types, both *in vitro* and *in vivo*^2^. Herein, we non-covalently conjugated ligand C_8_ to AS1411, LNA-AS1411 and U-AS1411 in an attempt to improve its biological properties (Table 1). Using a simple supramolecular strategy lacking any linkage conjugation, we tested a ligand delivery system aimed at HeLa cancer cells as schematically depicted in Fig. 1. The use of locked nucleic acids (LNA) or via thymine-to-uracil substitutions may enhance the binding affinity of the aptamer to its target nucleolin^12^. LNA nucleotides comprise a 2′-*O*, 4′-*C*-methylene linkage in the sugar moiety, locking it in a *N*-type conformation mimicking RNA nucleotide. LNAs display enhanced thermal and ribozyme stability, and low cytotoxicity for healthy cells^12^. dT → rU substitutions can contribute to the enhancement of stability due to loss of hydration leading to improved stacking of G-tetrads^13^.

**Table 1:** Nucleotide sequence of the aptamers used in this study and their spectroscopic profile characterization.

Aptamer	Sequence^a^	CD profile^b^		TDS factor^c^		
		240 nm	260 nm	$\frac{{\rm{\Delta }}{\rm{A}}240}{{\rm{\Delta }}{\rm{A}}295}$	$\frac{{\rm{\Delta }}{\rm{A}}255}{{\rm{\Delta }}{\rm{A}}295}$	$\frac{{\rm{\Delta }}{\rm{A}}275}{{\rm{\Delta }}{\rm{A}}295}$
AS1411	5′-GGTGGTGGTGGTTGTGGTGGTGGTGG-3′	−	+	2.42	1.51	1.84
LNA-AS1411	5′-***g***GTGGTGGTG***g***TTGTGGTGGTGGTG***g***-3′	−	+	2.87	3.32	2.90
U-AS1411	5′-GGTGGTGGTGG**UU**GTGGTGGTGGTGG-3′	−	+	2.17	2.04	2.13

^a^Modifications are highlighted in bold, ***g*** represents LNA guanine.

^b^“+” and “−” refer to the presence of a positive or negative peak, respectively, at the indicated wavelength.

^c^Ratio between values at two different wavelengths (*e.g*., 240 and 295 nm for the left column).

**Figure 2:**
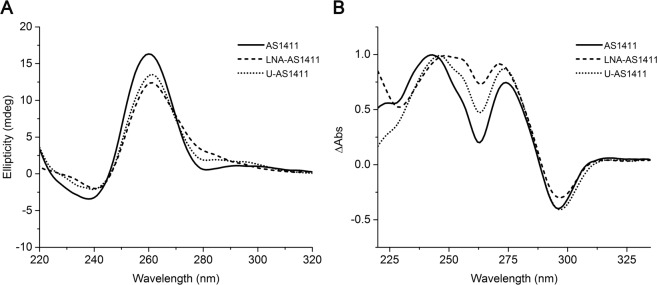
Circular dichroism spectra (**A**) and thermal difference spectra (**B**) of AS1411 aptamer and its derivatives LNA-AS1411 and U-AS1411. Spectra acquisition was performed in 30 mM potassium phosphate buffer containing 100 mM KCl.

The effect of the modifications on the overall G-quadruplex structure was evaluated by circular dichroism (CD) and UV/Vis spectroscopy, namely by the use of thermal difference spectra (TDS). The CD and TDS spectra of AS1411, LNA-AS1411 and U-AS1411 are presented in Fig. 2. The CD spectra of the three sequences, despite minor changes between them, show the typical CD signature of parallel G-quadruplex topologies (Fig. 2A), with a positive peak at around 260 nm and a negative peak at around 240 nm. However, as shown previously by Trent and collaborators, AS1411 forms a mixture of at least eight G-quadruplex species in solution and CD spectra could not discriminate among the different species^14^. The TDS spectra confirm the structural polymorphism inherent to the sequence (Fig. 2B). The positive peaks around 243 and 273 nm, and negative peak around 295 nm confirm that a G-quadruplex structure is adopted by the modified AS1411 aptamers^8^. Three TDS factors were determined and presented in Table 1. The TDS factor (ΔA_240_/ΔA_295_) appeared between 2.17 and 2.87, indicating a mixture of G-quadruplex topologies as a factor lower than 2 and higher than 4 is characteristic of antiparallel and parallel topologies, respectively^15^. ΔA_255_/ΔA_295_ factor on its turn, was between 1.51 for AS1411 and 3.32 for LNA-AS1411. Likewise, these values demonstrate structural polymorphism as a factor lower than 1.5 and higher than 3.5 is characteristic of antiparallel and parallel topologies, respectively^15^. Finally, ΔA_275_/ΔA_295_ factor was 1.84, 2.13 and 2.90 for AS1411, U-AS1411 and LNA-AS1411 respectively. Factors lower than 2 and higher than 3 are characteristic of antiparallel and parallel topologies, respectively^15^. AS1411 value is indicative of an antiparallel topology, despite the inherent polymorphism. This may be due to the fact that some of the possible topologies predicted for AS1411 are antiparallel dimers^14^.

The global analysis of the TDS factors shows that despite the evident structural polymorphism, LNA-AS1411 may fold preferentially into a parallel G-quadruplex as the ΔA_255_/ΔA_295_ and ΔA_275_/ΔA_295_ values are higher when compared with the other sequences, and closer to the threshold value for a parallel topology. Indeed, LNA guanosines are incompatible with a *syn* conformation which prevents their assembly into the anti-parallel conformation^16^. It has been proposed that the most stable parallel G- quadruplex is obtained when substituting every guanosine to the LNA except the 5′-terminal one^16^. The observation of the CD spectra shows the loss of a weak negative band at around 280 nm which may be indicative of the structural transitions^17^. Nonetheless, the modifications introduced in AS1411 sequence do not seem to promote the formation of a single G-quadruplex structure nor affect G-quadruplex formation.

**Figure 3:**
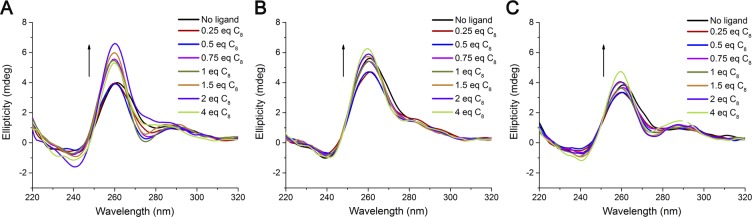
Circular dichroism spectra of (**A**) AS1411, (**B**) LNA-AS1411 and (**C**) U-AS1411 aptamers in the presence of increasing amounts of ligand C_8_. Spectra acquisition was performed in 30 mM potassium phosphate buffer containing 100 mM KCl.

The formation of a stable complex between the aptamers and ligand C_8_ was assessed and characterized by CD and fluorescence spectroscopy. CD titrations were first performed to assess complex formation and/or structural modification of the G-quadruplex structure upon ligand binding. The results are shown in Fig. 3.

Upon ligand titration, the overall topology was maintained followed by an increase in ellipticity indicating aptamer-ligand association and stabilization of the preformed G-quadruplex structure. A deeper analysis of Fig. 3A,C reveals significant changes in the 270–300 nm region. This may be due to the preferential stabilization of one of the possible topologies of AS1411 and U-AS1411. Such effect is not observed for LNA-AS1411 potentially due to the formation of parallel topologies only.

**Figure 4:**
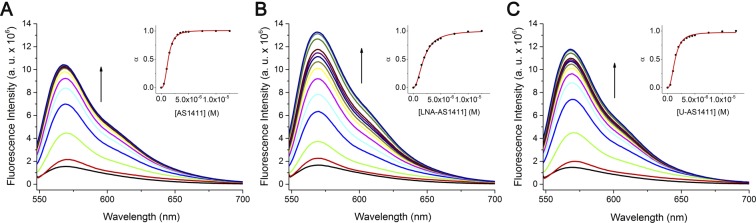
Fluorescence spectroscopy titrations of ligand C_8_ with increasing amounts of (**A**) AS1411, (**B**) LNA-AS1411 and (**C**) U-AS1411 DNA. Spectra acquisition was performed in 30 mM of potassium phosphate buffer containing 100 mM KCl, with DNA concentration ranging from 0.1 μM to 12.5 μM. Insets: saturation binding plots fitted to Hill model.

The binding strength between ligand C_8_ and the aptamers was further assessed using fluorimetric titrations. As seen in Fig. 4, upon excitation at 498 nm, ligand C_8_ emitted fluorescence as a broad band centred at 568 nm as previously reported^6^. After addition of previously annealed aptamer solution, a ≈10-fold fluorescence enhancement was observed for all sequences denoting a strong interaction between the ligand and the aptamers G4 structure. The fluorescence intensity values were then fitted to a saturation binding model and the apparent dissociation constants (*K*_D_) determined. The *K*_D_ values for AS1411, LNA-AS1411 and U-1411 were 1.29 × 10^−6^, 1.92 × 10^−6^ and 1.37 × 10^−6^ M, respectively (SD ≤ 1 × 10^−8^ M). These values in the low micromolar range are indicative of moderate to high affinity and stable complex formation between the ligand and the aptamers G4 structure^18^.

The Hill coefficient (*n*) which can be interpreted as the average number of bound ligands per G4, was 2.8, 2.1 and 2.5 for AS1411, LNA-AS1411 and U-1411, respectively. This suggests a ligand:DNA ratio of 3:1 in the case of AS1411 and U-1411, and 2:1 in the case of LNA-AS1411. Regarding AS1411, as it is assumed to fold into a variety of structures, for instance a dimeric antiparallel G-quadruplex^2^, the 3:1 ratio mas be misestimated. At this point, we have no information on whether ligand C_8_ binds preferentially to a specific AS1411 conformation. Additionally, the Hill coefficient is a measure of cooperativity, whereas the *n* values greater than 1 indicate positive cooperativity meaning that a binding event increases the affinity of additional ligand molecules^6^.

The influence of ligand C_8_ on the thermal stability of the aptamers was investigated by thermal denaturation. For that, we performed CD melting experiments and assessed the ligand’s effect on the aptamers melting temperature (*T*_m_) (Fig. S1). The ligand clearly increased the *T*_m_ values of all the aptamers G4 structure, being the effect more prominent on AS1411, as C_8_ promoted a Δ*T*_m_ of 28.4 °C at 2 molar equivalents. U-AS1411 was stabilized for 24.5 °C at the same ligand:G4 ratio, while LNA-AS1411 was the least stabilized structure with a Δ*T*_m_ of 17.5 °C. The Δ*T*_m_ values agree with the *K*_D_ values as the AS1411 presented the higher *K*_D_ and Δ*T*_m_ while on the other end LNA-AS1411 presented the lowest values. As the G4 folding is necessary for the biological properties of the aptamer, the ligand-induced thermal stability may enhance its nuclease resistance, nucleolin affinity and intracellular trafficking^12,19,20^. The Δ*T*_m_ values at different ligand:G4 ratios are summarized in Table S1.

The ability of the aptamers AS1411, LNA-AS1411 and U-AS1411 to accumulate C_8_ in the cancer cells and to prevent C_8_ accumulation in healthy cells was indirectly assessed by the MTT assay. As previously reported, AS1411 has a weak inhibitory effect in HeLa cancer cells and more than 5 days are needed to observe a slight effect on cell viability, even at 10 μM aptamer concentration^21,22^. As such, we tested the effect of the aptamer-ligand complexes for 7 days incubation period at 15 μM aptamer concentration. The free aptamers cytotoxicity towards HeLa and non-malignant NHDF cells is shown in Fig. S2. AS1411 showed reduced cytotoxicity towards HeLa cells (79% mean viability) and nearly no toxicity towards non-malignant cells. The LNA-modified aptamer did not have any effect on the cell viability of both cancer and non-malignant cells. Finally, U-AS1411 presented a similar cytotoxicity for HeLa cells (71% mean viability) as AS1411, however in the case of NHDF cells its cytotoxicity was significantly higher (53% mean viability).

**Figure 5:**
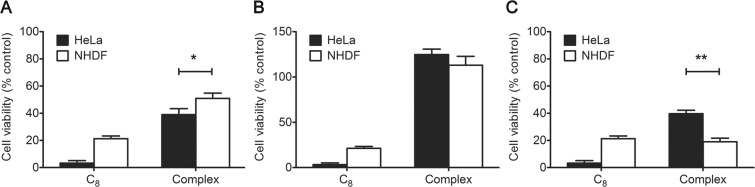
Relative cell viability of HeLa and NHDF cells incubated for 7 days with free C_8_ or the pre-formed aptamer-ligand complex at a C_8_ concentration of 1 μM using aptamer (**A**) AS1411, (**B**) LNA-AS1411 and (**C**) U-AS1411. *p < 0.05, **p < 0.01.

Then, the IC_50_ values of C_8_ at 7 days incubation time were determined for HeLa and NHDF cells (Fig. S3). The IC_50_ was 0.15 and 0.48 µM for HeLa and NHDF, respectively. Based on these values, we decided to use a C_8_ concentration of 1 µM which is high enough to elicit significant cell toxicity and still suitable to observe any changes induced by the ligand complexation to the aptamer. After mixing 15 µM aptamer with 1 µM C_8_ to allow complex formation, the aptamer-ligand complexes were incubated for 7 days in HeLa and NHDF cells. The results are presented in Fig. 5.

As seen in Fig. 5A, the C_8_ toxicity towards non-malignant cells is significantly attenuated upon complexation with AS1411. The lower cytotoxicity of C_8_ towards NHDF cells when complexed with AS1411 may be due to the aptamer mechanism involved in selective accumulation in cells. It has been proposed that AS1411 is gradually cleared from normal cells by efflux or exocytosis 24–72 h post-treatment^2^. Additionally, normal cells have increased lysosomal activity in comparison with AS1411-treated cancer cells^2^. Therefore, if the aptamer-ligand complex is capable of sustaining cellular trafficking it may be cleared from normal cells by lysosomal degradation, thus clearing C_8_ and reducing its effects. Although AS1411-C_8_ has a more pronounced effect on HeLa than on NHDF cells, the cytotoxicity towards HeLa cells is also hindered. In this case, the explanation may be the high affinity of C_8_ towards the aptamer which may prevent the ligand to detach from AS1411 once inside the cell. LNA-AS1411 complex on its hand, showed no cytotoxic effect on both cell lines with viability values around 100% (Fig. 5B). Indeed, LNA-containing DNA sequences were already shown to be poorly toxic for HeLa cells^23^. This suggests that the modification of the sugar ring may affect the aptamer cellular behaviour, namely the interaction with its target nucleolin. The apparent viability values above 100% observed for LNA-AS1411 treatment may be due to the compounds influencing the cell’s metabolism thus resulting in increased metabolization of MTT^24^ (as the MTT assay is a measure of the cellular mitochondrial enzymatic activity) and/or cell growth (in response to stress, a phenomena termed hormesis^25^) when compared with the control. Opposingly, U-AS1411 complex showed an undesired increased cytotoxicity towards NHDF cells, significantly higher than that observed for HeLa cells (Fig. 5C).

**Figure 6:**
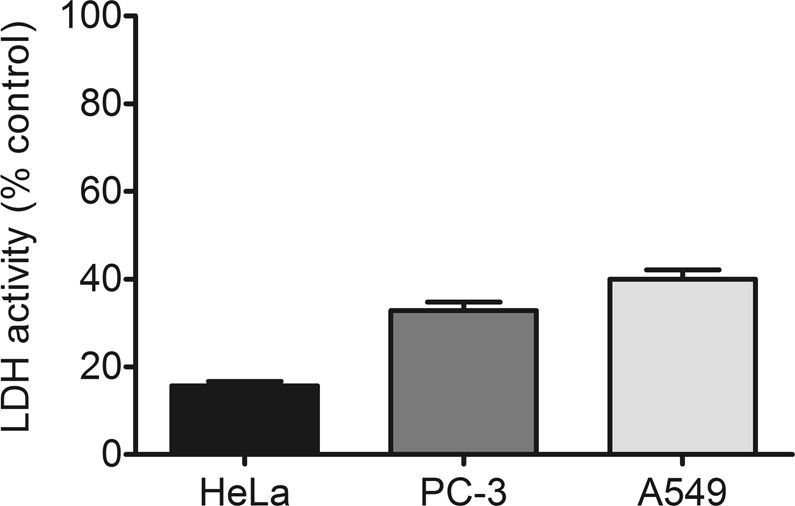
Relative LDH activity of HeLa, PC-3 and A549 cells incubated for 7 days with the pre-formed AS1411-ligand complex at a C_8_ concentration of 1 μM.

To show the applicability of the system, the MTT experiments were extended to two additional cancer cell lines using AS1411-C_8_ complex, namely PC-3 prostate cancer cell line and A549 lung cancer cell lines (Fig. S4). Similarly, to what was observed for HeLa cells, the aptamer cytotoxicity was improved when complexed with C_8_. In the case of PC-3 cells the complex promoted a viability decrease of about 85% while in the case of A549 cells a milder effect was observed (47% mean viability). These results suggest that this strategy may be employed in other cancer types, particularly prostate cancer because PC-3 cells showed the most promising results. Additionally, the effect of AS1411-C_8_ complex on cell membrane integrity was evaluated in the same cancer cell line panel used in the MTT assays. The results shown in Fig. 6 suggest that the AS1411-C_8_ complex promoted modest cell membrane damage in the tested cell lines (LDH activity <50%), despite reducing their viability as indicated by the MTT results. Similar effect was already observed for other nanosystems^26^ and may be related to a non-apoptotic cell death mechanism^27^. Furthermore, it should be emphasized that while the LDH assay is a measure of cytotoxicity induced by cell membrane damage, the MTT assay provides data on cell viability, specifically the cell’s metabolic activity. A correlation between the results is therefore not always attained.

**Figure 7:**
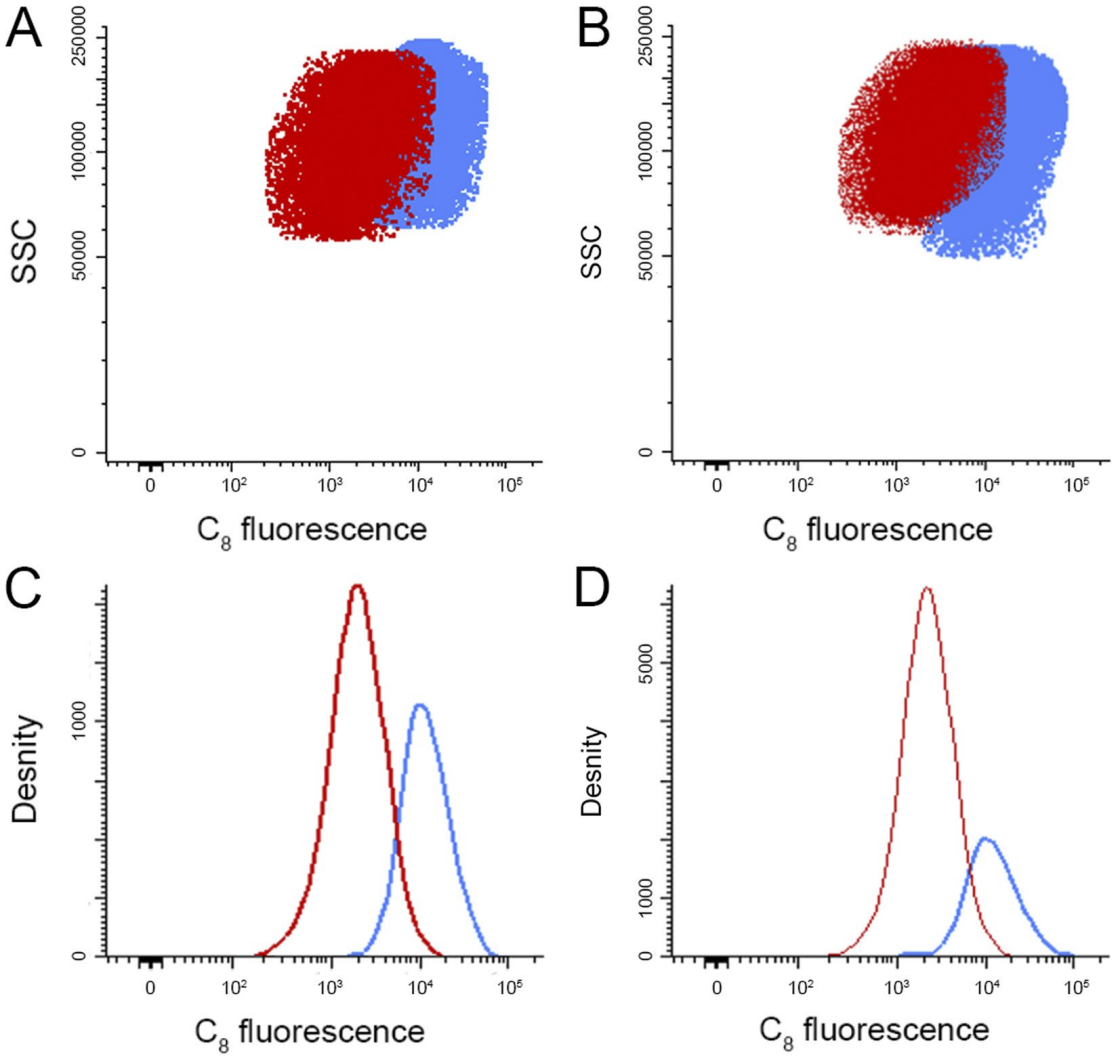
Flow cytometry analysis of free C_8_ (blue) and AS1411-C_8_ complex (red) in (**A**) HeLa cancer cells and (**B**) NHDF non-malignant cells and respective histograms (**C**,**D**). Cells were incubated for 24 h with the preformed aptamer-ligand complex at a C_8_ concentration of 1 μM.

Following the MTT and LDH assays, we employed flow cytometry to assess intracellular accumulation of free and AS1411-complexed C_8_ in HeLa cancer cells and NHDF non-malignant cells, based on the intrinsic fluorescence of ligand C_8_. The results are depicted in Fig. 7.

Flow cytometry analysis indicated that the AS1411-C_8_ complex is less intracellularly accumulated in both cell types when compared to free C_8_, as observed by the lower fluorescence intensity emitted from the cells, despite an enhanced fluorescence quantum yield of C_8_ when complexed to DNA. These results corroborate the lower cytotoxicity observed for the AS1411-C_8_ complex when compared with free C_8_.

**Figure 8:**
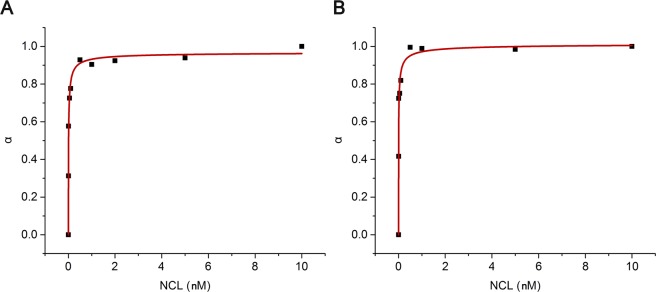
Saturation binding plots fitted to Michaelis-Menten model. Fluorescence intensity of Cy-AS1411 was recorded after the addition of increasing amounts of nucleolin in the (**A**) absence and (**B**) presence of ligand C_8_.

Then, we assessed if the reduced cellular uptake of the aptamer-ligand complex could be due to a potential interference with nucleolin binding. For that we employed fluorescence spectroscopy in two different approaches. First, using a solution of fluorescently labelled Cy3-AS1411, we titrated increasing amounts of nucleolin in the presence and absence of C_8_ and determined the *K*_D_ values from the fluorescence intensity changes. The saturation binding plots are shown in Fig. 8. The obtained *K*_D_ values for the binding interaction between the free AS1411 aptamer and the AS1411-C_8_ complex with nucleolin were 8.63 pM and 6.38 pM, respectively. The obtained *K*_D_ values are lower than those reported in the literature, being potentially overestimated by the use of labelled oligonucleotides or the use of a different peptide than that used in those studies^28,29^. Nonetheless, both values are in the same range, being slightly lower in the case of the aptamer-ligand complex which suggests that the ligand does not negatively affect the recognition of the protein by the aptamer.

Additionally, a competition FRET-melting experiment was conducted to support the fluorescence titration findings. The rationale for the assay design was based on the hypothesis that if the ligand-induced stabilization of AS1411 was similar in the presence and absence of nucleolin, ligand binding would not affect protein recognition. The ligand-induced thermal stabilization of AS1411 was 13.1 °C in the presence of 1 µM C_8_ (Table 2). Upon pre-incubation of AS1411 with equimolar nucleolin, the Δ*T*_m_ increased to 20.0 °C. Even with an excess of protein (2 molar eq.) the Δ*T*_m_ was 15.1 °C, reflecting a higher stabilization of the aptamer by C_8_ in the presence of nucleolin. This indicates that the ligand does not prevent the recognition of the protein by the aptamer, as suggested by the fluorescence titration experiments, and that it may even enhance the stabilization of AS1411 G-quadruplex structure. This may be due to the ligand providing additional binding sites for the protein interaction (ternary complex), or since the aptamer G-quadruplex structure is necessary for nucleolin binding, the C_8_-induced stabilization of the structure may enhance its binding strength^30^.

**Table 2:** Ligand induced thermal stabilization of AS1411 aptamer in the presence and absence of nucleolin.

	Δ*T*_m_ AS1411 (°C)	Δ*T*_m_ AS1411 + 1 μM nucleolin (°C)	Δ*T*_m_ AS1411 + 2 μM nucleolin (°C)
C_8_	13.1 ± 0.8	20.0 ± 1.4	15.1 ± 0.7

^a^Δ*T*_m_ represents the difference in melting temperature [Δ*T*_m_ = *T*_m_ (DNA + 1 μM of ligand) − *T*_m_ (DNA)]. The buffer used was 10 mM lithium cacodylate, pH 7.2, supplemented with 10 mM KCl and 90 mM LiCl. The *T*_m_ value for AS1411 is 46.9 ± 0.2 °C. Reported values correspond to the average of three measurements with the estimated standard deviation.

**Figure 9:**
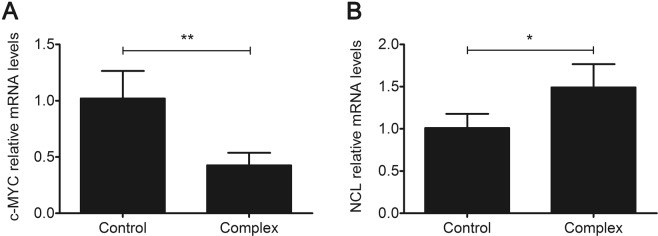
Effect of AS1411-C_8_ complex treatment on c-MYC and nucleolin (NCL) gene expression measured by RT-qPCR. Data represent the average of three experiments ± SEM; *p < 0.05, **p < 0.01.

To assess the potential effect of the AS1411-C_8_ complex on gene expression, RT-qPCR analysis was performed. Oncogene c-MYC and nucleolin mRNA levels were analysed since c-MYC is commonly found upregulated in tumour cells being responsible for cancer cell proliferation^31^, and nucleolin is the AS1411’s target. The results are depicted in Fig. 9. Upon treatment with AS1411-C_8_ complex, the expression of c-MYC mRNA was downregulated by more than 50% (p < 0.01) which may inhibit cancer cell progression, eventually leading to cell death^31^. The acridine derivative C_8_ was already tested for its ability to stabilize the G4 structure formed by c-MYC NHE element of the promoter region^6^. Therefore, it is reasonable to assume that the decrease in the c-MYC mRNA levels may be due to transcription modulation induced by interaction of C_8_ and c-MYC G4 structures upon intracellular dissociation from the AS1411-ligand complex^32^. Opposingly, nucleolin mRNA expression was upregulated by 1.5-fold similarly to what was reported for free AS1411 before^33^. This suggests that the biological effects of AS1411 are not negatively affected by its complexation with ligand C_8_. This feedback loop induction may be responsible for increasing the cells sensitivity to AS1411 and/or increasing its intracellular accumulation^33^.

**Figure 10:**
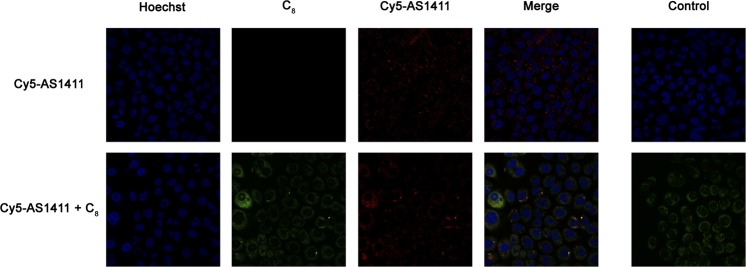
Confocal microscopy images of HeLa cells incubated with Cy5-AS1411 and Cy-AS1411-C_8_ complex for 7 days. Cell nuclei are stained with Hoechst 33342 (blue), C_8_ emits green fluorescence and Cy-AS1411 is shown in red. Overlapping of the two stains, observed as yellow regions, can be seen in the merged images of the cells. Control cells and C_8_ single stain control images are shown on the right. Brightness was adjusted to facilitate analysis.

Finally, the subcellular localization of the AS1411-C_8_ complex in HeLa and NHDF cells was evaluated by fluorescence confocal microscopy. As seen in Fig. 10, the complex is able to penetrate cell membrane and localizes in the cytoplasm of HeLa cells. The overlapping of C_8_ (green) and AS1411 (red) suggests that the complex is still formed intracellularly (yellow) and maintained during cell internalization and trafficking. C_8_ can be seen in the nucleoli in a free state which may suggest decomplexation and localization of the nucleolus as described for the ligand^6^. This may be particularly important to explain the downregulation of c-MYC transcriptional level as showed by the RT-qPCR experiments. A modest increase in the uptake of AS1411 can be observed in the presence of C_8_ which may correlate with the proposed increased stability of aptamer upon ligand binding, possibly enhancing its association with nucleolin, and the fact that nucleolin is found overexpressed upon treatment with the complex as suggested by RT-qPCR results. The quantitative analysis of the Cy5-AS1411 fluorescence in the absence and presence of C_8_ shown in Fig. S5 substantiates an increased uptake of AS1411 in the presence of the ligand.

**Figure 11:**
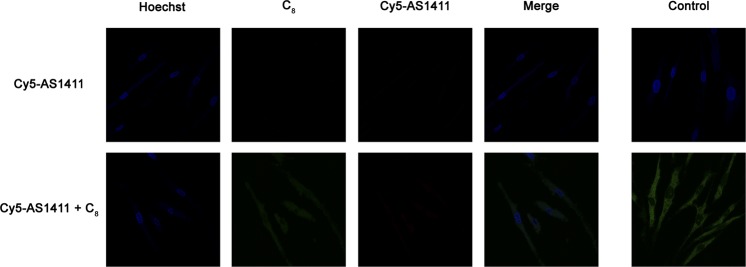
Confocal microscopy images of NHDF cells incubated with Cy5-AS1411 and Cy-AS1411-C_8_ complex for 6 days followed by 1-day incubation in fresh media. Cell nuclei are stained with Hoechst 33342 (blue), C_8_ emits green fluorescence while Cy5-AS1411 fluorescence is absent. Control cells and C_8_ single stain control images are shown on the right. Brightness was adjusted to facilitate analysis.

Similar findings were observed for NHDF cells (Fig. S6). These results disagree with the selective accumulation described above where it has been shown that AS1411 is gradually cleared from normal cells by efflux 24–72 h post-treatment. The explanation may arise from the fact that cells are constantly incubated in media containing the compounds for 7 days straight. To test this hypothesis, we incubated the cells for 6 days in the presence of the aptamer-ligand complex, followed by 1 day in free media. The results shown in Fig. 11 confirm that indeed AS1411 is eliminated from normal cells after one day. C_8_ despite being uptaken complexed with AS1411, remains inside the cells, suggesting that the observed differences in cytotoxicity in MTT assays between free C_8_ and AS1411-C_8_ complex are mostly due to differences in uptake as shown by flow cytometry. A similar experiment was performed in HeLa cells (Fig. S7) and the results suggest that cancer cells are indeed less efficient in eliminating the aptamer in its free or C_8_-complexed state as Cy5-AS1411 red staining is still observed after 1-day incubation in free media. These results agree with the proposed mechanism of cancer-selective accumulation of AS1411 where AS1411-conjugates are able to escape lysosomal vesicles with increased uptake^2,34^.

## Conclusion

AS1411 aptamer has been increasingly suggested as an effective targeting system for the selective delivery of a wide range of cargoes to cancer cells. Herein, using a simple supramolecular strategy lacking any chemical modification of the aptamer, we propose a delivery system for conveying a G-quadruplex ligand C_8_ into cancer cells in a selective manner. AS1411 unmodified aptamer and two derivatives containing LNA and uracil nucleotides were used. Ligand C_8_ was shown to strongly associate with the three aptamers with *K*_D_ in the 10^−6^ M range. Additionally, C_8_ is able to thermally stabilize the aptamers structure, possible enhancing its properties. The MTT assays shown that the complexation of C_8_ with AS1411 decreases its cytotoxicity to NHDF cells, which was later revealed to be due to differential uptake of free C_8_ vs complexed C_8_ by flow cytometry analysis. LNA-AS1411 and U-AS1411 complexes shown non-adequate cytotoxic profile, being that the first is non-toxic for HeLa cells and the latter is highly toxic for non-malignant cells. MTT experiments with prostate and lung cancer cells show the potential applicability of the system in other cancer types. Using fluorescence spectroscopy and FRET-melting we propose that ligand C_8_ complexation with AS1411 does not affect its recognition of protein nucleolin, which is necessary for its biological effects. RT-qPCR experiments demonstrated that AS1411-C_8_ complex is able to downregulate c-MYC expression, which may be attributed to the stabilization of c-MYC G4 structures by the ligand. Finally, confocal microscopy indicated that the complex is efficiently taken up into the cells and that AS1411-C_8_ is maintained during cell internalization and trafficking. Moreover, AS1411 uptake is increased in the presence of C_8_ which may be due to nucleolin overexpression induced by the complex as suggested by RT-qPCR. This study paves the way for the development of cancer-specific AS1411-based delivery systems for the selective accumulation of G-quadruplex ligands in the target tissues, in a simple non-covalent strategy.

## Supplementary information

Supplementary Information

## References

[1] Bates PJ, Laber Da, Miller DM, Thomas SD, Trent JO (2009). Discovery and development of the G-rich oligonucleotide AS1411 as a novel treatment for cancer. Exp. Mol. Pathol..

[2] Bates PJ (2017). G-quadruplex oligonucleotide AS1411 as a cancer-targeting agent: Uses and mechanisms. Biochim. Biophys. Acta - Gen. Subj..

[3] Mongelard F, Bouvet P (2007). Nucleolin: a multiFACeTed protein. Trends Cell Biol..

[4] Reyes-Reyes EM, Šalipur FR, Shams M, Forsthoefel MK, Bates PJ (2015). Mechanistic studies of anticancer aptamer AS1411 reveal a novel role for nucleolin in regulating Rac1 activation. Mol. Oncol..

[5] Shieh YA, Yang SJ, Wei MF, Shieh MJ (2010). Aptamer-based tumor-targeted drug delivery for photodynamic therapy. ACS Nano.

[6] Carvalho J (2018). Fluorescent light-up acridine orange derivatives bind and stabilize KRAS-22RT G-quadruplex. Biochimie.

[7] Pereira E (2017). Evaluation of Acridine Orange Derivatives as DNA-Targeted Radiopharmaceuticals for Auger Therapy: Influence of the Radionuclide and Distance to DNA. Sci. Rep..

[8] Mergny J-L, Li J, Lacroix L, Amrane S, Chaires JB (2005). Thermal difference spectra: A specific signature for nucleic acid structures. Nucleic Acids Res..

[9] Kumar, P., Nagarajan, A. & Uchil, P. D. Analysis of Cell Viability by the Lactate Dehydrogenase Assay. *Cold Spring Harb. Protoc*. 465–469, 10.1101/pdb.prot095497 (2018). 10.1101/pdb.prot095497 29858337

[10] De Rache A, Mergny J-LL (2015). Assessment of selectivity of G-quadruplex ligands via an optimised FRET melting assay. Biochimie.

[11] Loureiro A (2018). Absence of Albumin Improves *in Vitro* Cellular Uptake and Disruption of Poloxamer 407-Based Nanoparticles inside Cancer Cells. Mol. Pharm..

[12] Gao, S., Zheng, X., Jiao, B. & Wang, L. Post-SELEX optimization of aptamers. *Anal. Bioanal. Chem*. 4567–4573, 10.1007/s00216-016-9556-2 (2016). 10.1007/s00216-016-9556-2 27173394

[13] Olsen, C. M. & Marky, L. A. Energetic and hydration contributions of the removal of methyl groups from thymine to form uracil in G-quadruplexes. *J. Phys. Chem. B*, 10.1021/jp808526d (2009). 10.1021/jp808526d 19198041

[14] Dailey MM, Clarke Miller M, Bates PJ, Lane AN, Trent JO (2010). Resolution and characterization of the structural polymorphism of a single quadruplex-forming sequence. Nucleic Acids Res..

[15] Karsisiotis AI (2011). Topological characterization of nucleic acid G-quadruplexes by UV absorption and circular dichroism. Angew. Chemie - Int. Ed..

[16] Pedersen EB, Nielsen JT, Nielsen C, Filichev VV (2011). Enhanced anti-HIV-1 activity of G-quadruplexes comprising locked nucleic acids and intercalating nucleic acids. Nucleic Acids Res..

[17] Bing, T. *et al*. Triplex-quadruplex structural scaffold: A new binding structure of aptamer. *Sci. Rep*., 10.1038/s41598-017-15797-5 (2017). 10.1038/s41598-017-15797-5 PMC5684193 29133961

[18] Lecarme, L. *et al*. Interaction of Polycationic Ni (II)-Salophen Complexes with G-Quadruplex DNA. (2014). 10.1021/ic502063r 25383703

[19] Hasegawa, H., Savory, N., Abe, K. & Ikebukuro, K. Methods for improving aptamer binding affinity. *Molecules* **21** (2016). 10.3390/molecules21040421 PMC6273865 27043498

[20] D’Onofrio J (2007). 5′-Modified G-quadruplex forming oligonucleotides endowed with anti-HIV activity: Synthesis and biophysical properties. Bioconjug. Chem..

[21] Bates PJ, Kahlon JB, Thomas SD, Trent JO, Miller DM (1999). Antiproliferative activity of G-rich oligonucleotides correlates with protein binding. J. Biol. Chem..

[22] Girvan AC (2006). AGRO100 inhibits activation of nuclear factor-Œ∫B (NF-Œ∫B) by forming a complex with NF-Œ∫B essential modulator (NEMO) and nucleolin. Mol. Cancer Ther..

[23] Arzumanov, A. *et al*. Inhibition of HIV-1 Tat-dependent trans activation by steric block chimeric 2′-O-methyl/LNA oligoribonucleotides. *Biochemistry* **40**, 14645–54 (2001). 10.1021/bi011279e 11724578

[24] Rai Y (2018). Mitochondrial biogenesis and metabolic hyperactivation limits the application of MTT assay in the estimation of radiation induced growth inhibition. Sci. Rep..

[25] Yoshimasu T (2015). A theoretical model for the hormetic dose-response curve for anticancer agents. Anticancer Res..

[26] Ursini CL (2012). Study of Cytotoxic and Genotoxic Effects of Hydroxyl-Functionalized Multiwalled Carbon Nanotubes on Human Pulmonary Cells. J. Nanomater..

[27] Kornienko A, Rastogi SK, Lefranc F, Kiss R (2013). Therapeutic Agents Triggering Nonapoptotic Cancer Cell Death. J. Med. Chem..

[28] Fan X, Sun L, Wu Y, Zhang L, Yang Z (2016). Bioactivity of 2′-deoxyinosine-incorporated aptamer AS1411. Sci. Rep..

[29] Fan X (2017). The Bioactivity of D-/L-Isonucleoside- and 2′-Deoxyinosine-Incorporated Aptamer AS1411s Including DNA Replication/MicroRNA Expression. Mol. Ther. - Nucleic Acids.

[30] Dapić V (2003). Biophysical and biological properties of quadruplex oligodeoxyribonucleotides. Nucleic Acids Res..

[31] Hurley LH, Von Hoff DD, Siddiqui-Jain A, Yang D (2006). Drug Targeting of the c-MYC Promoter to Repress Gene Expression via a G-Quadruplex Silencer Element. Semin. Oncol..

[32] Chauhan A (2016). Synthesis of Fluorescent Binaphthyl Amines That Bind c-MYC G-Quadruplex DNA and Repress c-MYC Expression. J. Med. Chem..

[33] Sharma VR (2018). Nucleolin Overexpression Confers Increased Sensitivity to the Anti-Nucleolin Aptamer, AS1411. Cancer Invest..

[34] Reyes-Reyes EM, Teng Y, Bates PJ (2010). A new paradigm for aptamer therapeutic AS1411 action: Uptake by macropinocytosis and its stimulation by a nucleolin-dependent mechanism. Cancer Res..

