# Supplementary material for: Aptamer-based Targeted Delivery of a G-quadruplex Ligand in Cervical Cancer Cells
Source: Sci Rep. 2019 May 28;9:7945. doi: 10.1038/s41598-019-44388-9 (PMC6538641; doi:10.1038/s41598-019-44388-9)
Supplement: Supplementary file 1 — Supplementary Information [file 41598_2019_44388_MOESM1_ESM.docx]

Supplementary Information

**Aptamer-based Targeted Delivery of a G-quadruplex Ligand in Cervical Cancer Cells**

Josué Carvalho ^1^, Artur Paiva ^2,3,4^, Maria Paula Cabral Campello ^5^, António Paulo ^5^, Jean-Louis Mergny ^6,7^, Gilmar F. Salgado ^6^, João A. Queiroz ^1^, Carla Cruz ^1,^*

^1^ CICS-UBI - Centro de Investigação em Ciências da Saúde, Universidade da Beira Interior, Av. Infante D. Henrique, 6200-506 Covilhã, Portugal. Tel: +351 275 329 076; Fax: +351 275 329 099.

^2^ Unidade de Gestão Operacional em Citometria, Centro Hospitalar e Universitário de Coimbra (CHUC), Portugal.

^3^ CIMAGO/iCBR/CIBB, Faculdade de Medicina da Universidade de Coimbra, Portugal.

^4^ Instituto Politécnico de Coimbra, ESTESC-Coimbra Health School, Ciências Biomédicas Laboratoriais, Portugal

^5^ Centro de Ciências e Tecnologias Nucleares, Instituto Superior Técnico, Universidade de Lisboa, Estrada Nacional 10 (km 139,7), 2695-066 Bobadela LRS, Portugal.

^6^ Univ. Bordeaux, ARNA laboratory, INSERM, U1212, CNRS UMR 5320, IECB, F-33600 Pessac, France.

^7^ Institute of Biophysics, AS CR, v.v.i. Kralovopolska 135, 612 65 Brno, Czech Republic.

* corresponding author email: carlacruz@fcsaude.ubi.pt

| **Table S1.** Ligand-induced thermal stabilization (∆*T*_m_) measured by CD melting experiments. | | | | | | |
| --- | --- | --- | --- | --- | --- | --- |
|  | | | ∆*T*_m_ (°C) ^a^ | | | |
| **Aptamer** |  | 0.5 eq C_8_ | | 1 eq C_8_ | 2 eq C_8_ | 4 eq C_8_ |
| **AS1411** |  | 12.7 ± 0.3 | | 20.5 ± 0.2 | 28.4 ± 0.2 | >30 |
| **LNA-AS1411** |  | 6.4 ± 0.2 | | 12.0 ± 0.2 | 17.5 ± 0.1 | 22.6 ± 0.2 |
| **U-AS1411** |  | 5.1 ± 0.5 | | 14.9 ± 0.5 | 24.5 ± 0.4 | >30 |
| ^a^ Δ*T*_m_ represents the difference in melting temperature [ Δ*T*_m_ = *T*_m_ (DNA + ligand) − *T*_m_ (DNA)]. The buffer used was 10 mM lithium cacodylate, pH 7.2, supplemented with 10 mM KCl and 90 mM LiCl. The *T*_m_ values for the aptamers are 56.3 ± 0.1 °C for AS1411, 51.6 ± 0.2 °C for LNA-AS1411, 52.6 ± 0.2 °C for U-AS1411. Values are reported as Δ*T*_m_ ± SE. | | | | | | |


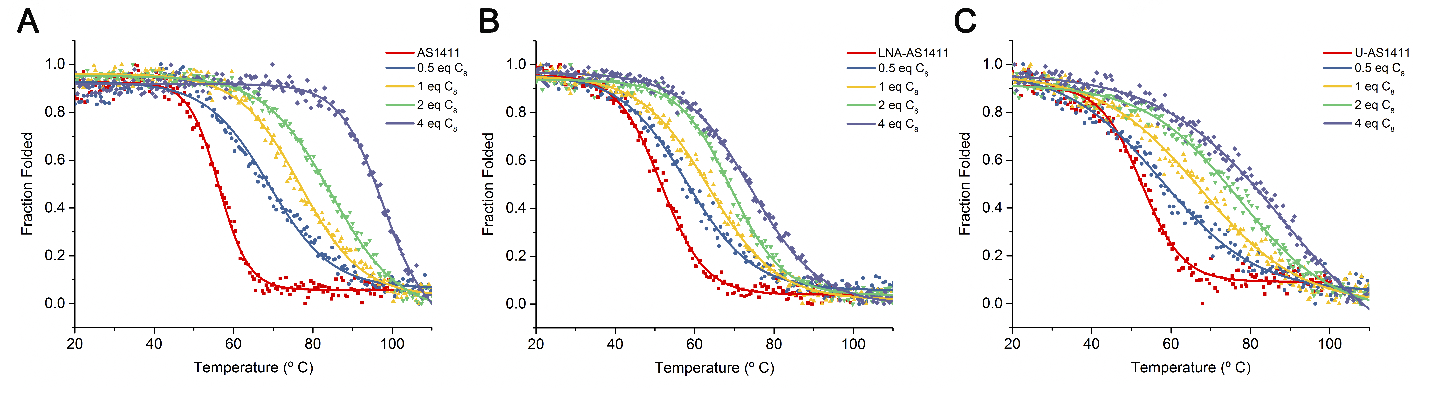


**Figure S1**. CD melting curves of (A) AS1411, (B) LNA-AS1411 and (C) U-AS1411 in the presence of increasing amounts of ligand C_8_. Melting curves were obtained by monitoring the ellipticity at 262 nm between 20 and 110 °C.


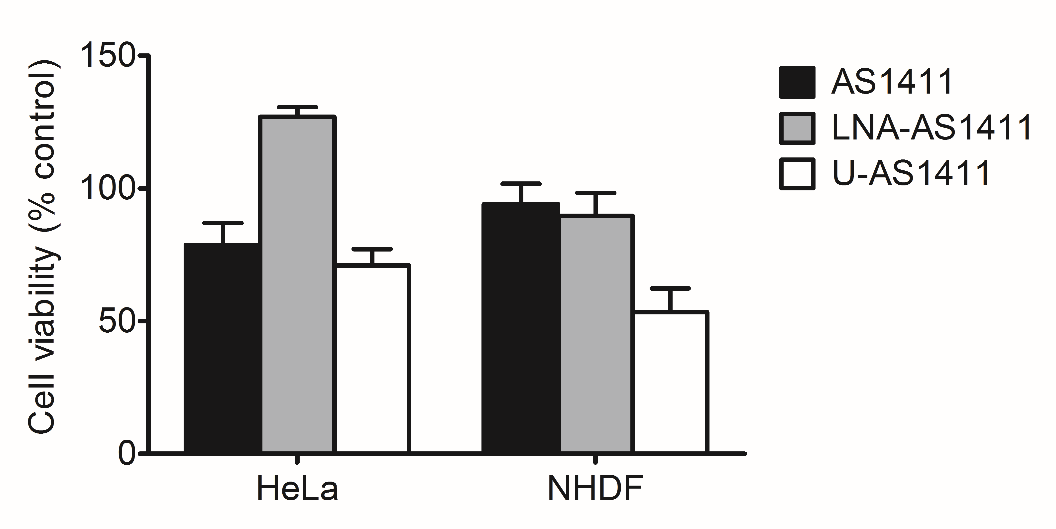


**Figure S2**. Relative cell viability of HeLa and NHDF cells incubated for 7 days with the aptamers at 15 μM.


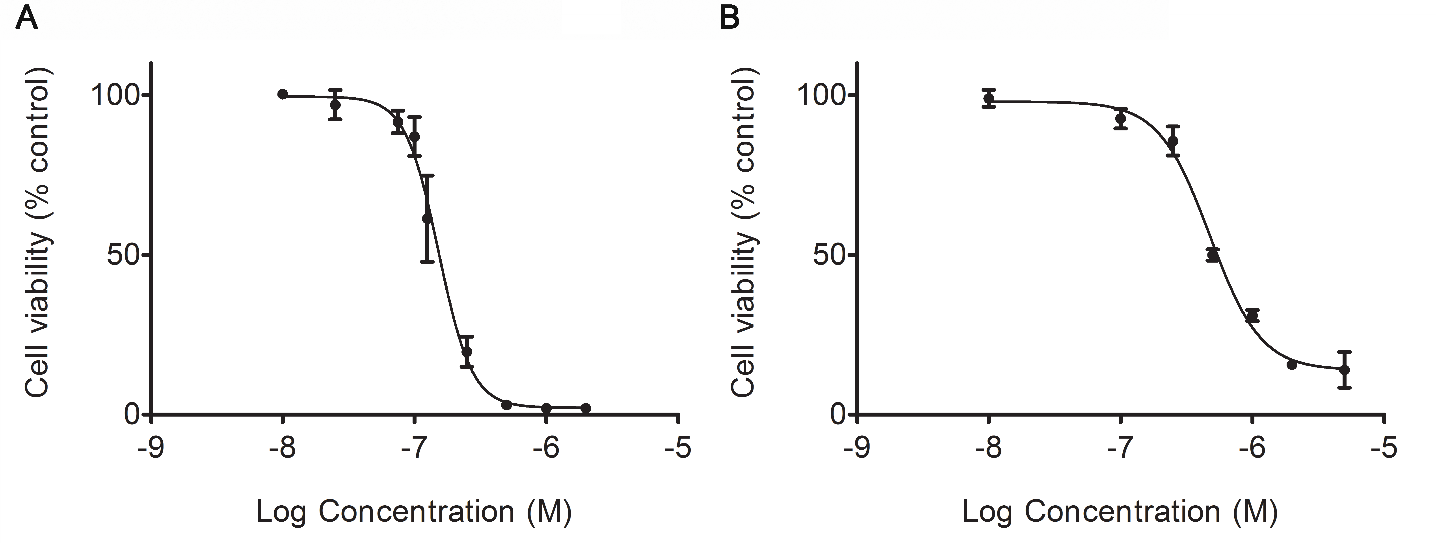


**Figure S3**. Dose-response data of cell viability measured by MTT assay after incubation of (A) HeLa and (B) NHDF cells with C_8_ for 7 days.


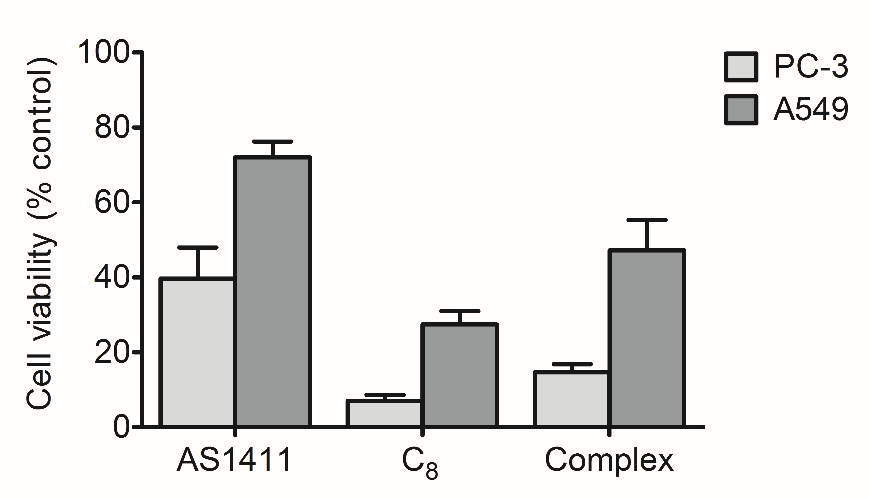


**Figure S4.** Relative cell viability of PC-3 and A549 cancer cells incubated for 7 days with free AS1411, free C_8_ or the pre-formed aptamer-ligand complex at a C_8_ concentration of 1 μM.


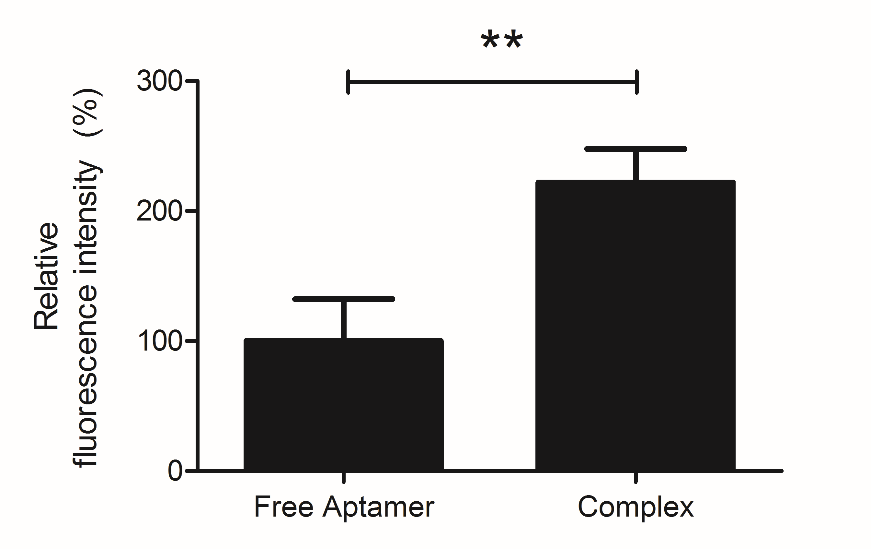


**Figure S5.** CTCF of the cells incubated with free Cy5-AS1411 or Cy5-AS1411-C_8_ complex. Data is represented as fluorescence intensity relatively to free aptamer images (positive control). Data represent the average of three experiments ± SEM, having considered at least 100 Cy5-AS1411-positive cells; ***p* < 0.01.


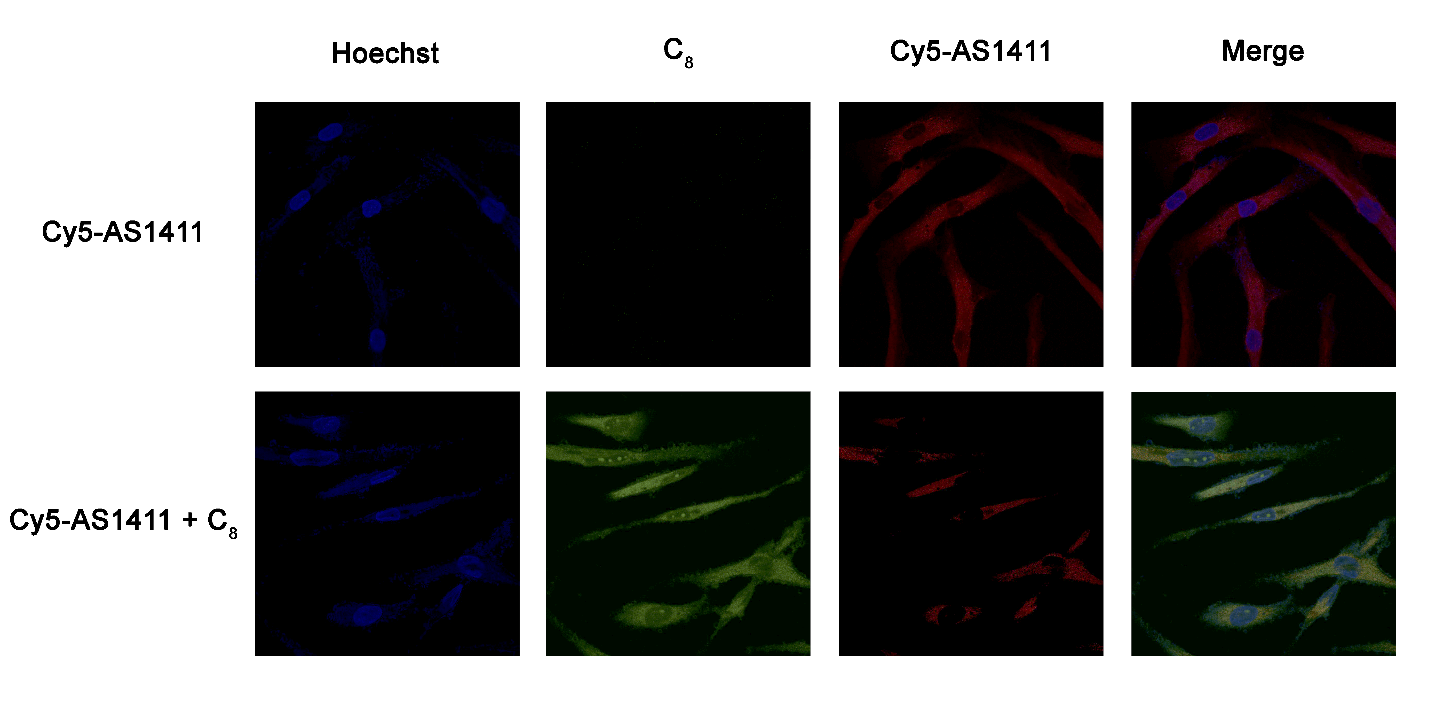


**Figure S6.** Confocal microscopy images of NHDF cells incubated with Cy5-AS1411 and Cy-AS1411-C_8_ complex for 7 days. Cell nuclei are stained with Hoechst 33342 (blue), C_8_ emits green fluorescence and Cy5-AS1411 is shown in red. Overlapping of the two stains, observed as yellow regions can be seen in the merge images. Brightness was adjusted to facilitate analysis.


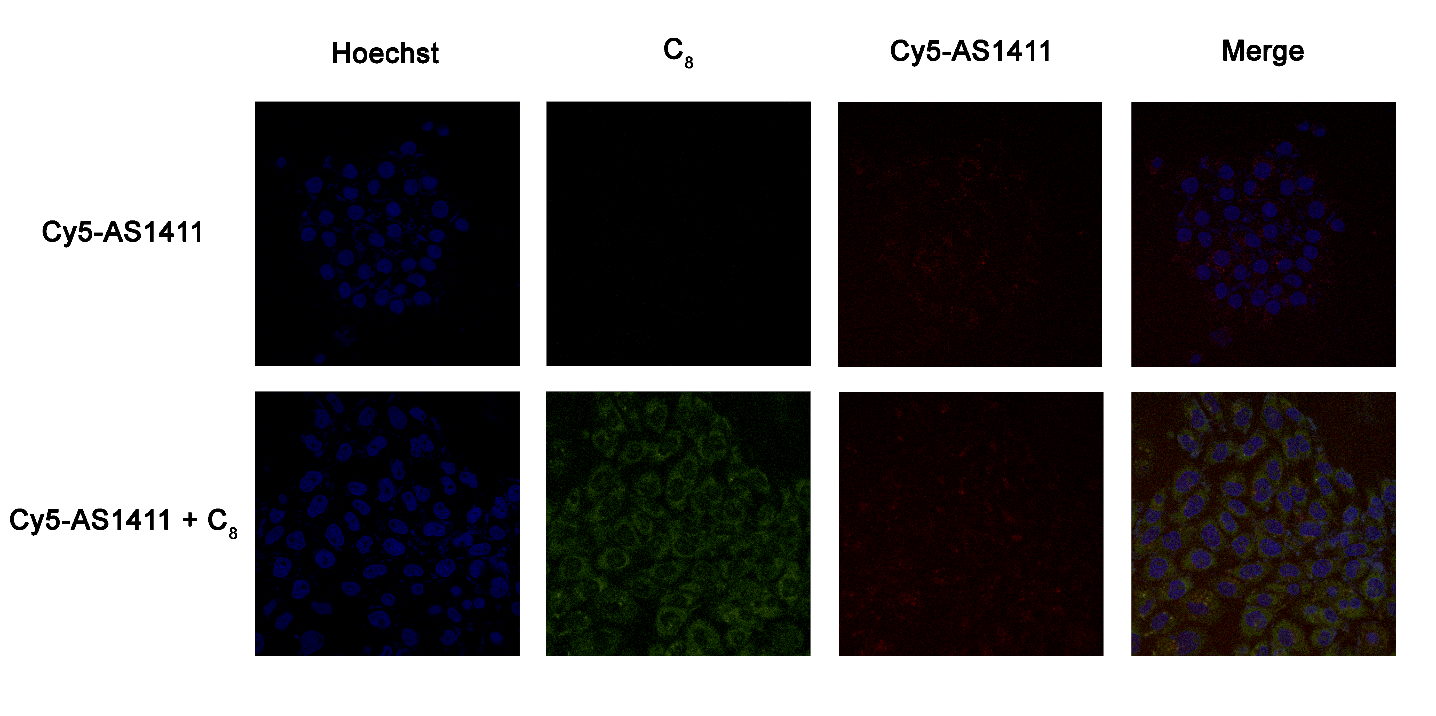


**Figure S7.** Confocal microscopy images of HeLa cells incubated with Cy5-AS1411 and Cy-AS1411-C_8_ complex for 6 days followed by 1-day incubation in fresh media. Cell nuclei are stained with Hoechst 33342 (blue), C_8_ emits green fluorescence while Cy5-AS1411 fluorescence is showed in red. Brightness was adjusted to facilitate analysis.
